# Autophagic Degradation Is Involved in Cell Protection against Ricin Toxin

**DOI:** 10.3390/toxins15050304

**Published:** 2023-04-23

**Authors:** Yu Wu, Clémence Taisne, Nassim Mahtal, Alison Forrester, Marion Lussignol, Jean-Christophe Cintrat, Audrey Esclatine, Daniel Gillet, Julien Barbier

**Affiliations:** 1 Université Paris-Saclay, CEA, INRAE, Département Médicaments et Technologies pour la Santé (DMTS), SIMoS, Gif-sur-Yvette 91191, France; 2 Institute of Immunology, The First Affiliated Hospital of USTC, Division of Life Sciences and Medicine, University of Science and Technology of China (USTC), Hefei 230001, China; 3 Université Paris-Saclay, CEA, CNRS, Institute for Integrative Biology of the Cell (I2BC), Gif-sur-Yvette 91198, France; 4 Research Unit of Biochemistry and Cell Biology (URBC), Namur Research Institute for Life Sciences (NARILIS), University of Namur, 5000 Namur, Belgium; 5 Université Paris-Saclay, CEA, INRAE, Médicaments et Technologies pour la Santé (DMTS), SCBM, Gif-sur-Yvette 91191, France

**Keywords:** ricin toxin, autophagy, degradation, ATG5, SMER28

## Abstract

Autophagy is a complex and highly regulated degradative process, which acts as a survival pathway in response to cellular stress, starvation and pathogen infection. Ricin toxin is a plant toxin produced by the castor bean and classified as a category B biothreat agent. Ricin toxin inhibits cellular protein synthesis by catalytically inactivating ribosomes, leading to cell death. Currently, there is no licensed treatment for patients exposed to ricin. Ricin-induced apoptosis has been extensively studied; however, whether its intoxication via protein synthesis inhibition affects autophagy is not yet resolved. In this work, we demonstrated that ricin intoxication is accompanied by its own autophagic degradation in mammalian cells. Autophagy deficiency, by knocking down ATG5, attenuates ricin degradation, thus aggravating ricin-induced cytotoxicity. Additionally, the autophagy inducer SMER28 (Small Molecule Enhancer of Rapamycin 28) partially protects cells against ricin cytotoxicity, an effect not observed in autophagy-deficient cells. These results demonstrate that autophagic degradation acts as a survival response of cells against ricin intoxication. This suggests that stimulation of autophagic degradation may be a strategy to counteract ricin intoxication.

## 1. Introduction

Ricin toxin is a highly potent toxin found in the seed of the plant *Ricinus communis*. It exerts its deleterious effect by inhibiting cellular protein synthesis [1]. Because of its relative ease of accessibility and production, it is considered a potential bioterrorism agent. Ricin is a globular, glycosylated heterodimer protein of approximately 60–65 kDa. It is a type II ribosome-inactivating protein (RIP) belonging to the class of AB toxins. Its structure is made of two different chains, named the ricin toxin A chain (RTA) and B chain (RTB), connected by a disulfide bond. RTA is a N-glycoside hydrolase composed of 267 amino acids. RTB is a lectin composed of 262 amino acids that is capable of binding terminal galactose residues on cell surface glycoproteins and glycolipids, which induces endocytosis of the toxin and trafficking to the endoplasmic reticulum (ER) [1]. In the lumen of the ER, similar to other AB toxins (e.g., Shiga toxin, *Pseudomonas* exotoxin, Cholera toxin), ricin avoids ER-associated degradation (ERAD) [2,3]. RTA uses the translocon Sec61 to translocate into the cytosol after the reduction of its interchain disulfide bond by the protein disulfide isomerase [4]. There, it depurinates an adenine on the sarcin ricin loop of the eukaryotic 28S ribosomal RNA in the 60S ribosomal subunit (adenine 4324 of rat 28S ribosomal RNA), leading to protein synthesis inhibition [5,6]. Moreover, ricin can activate cell stress responses and induce cell death via apoptosis [7]. Ricin can act on DNA directly or indirectly by inhibiting a DNA repair pathway and increasing reactive oxygen species (ROS) production [3]. Thus, ricin-induced cell damage normally occurs through multiple pathways [3,8]. The primary manifestations of intoxication by ricin depend on the route of entry: aerial, oral or parenteral. They are characterized by respiratory distress and hemoptysis in cases of inhalation; abdominal pain, vomiting and bloody diarrhea in case of ingestion; and local inflammation, pain and necrotic cellulitis in cases of injection. In the following hours or days, the toxin diffuses in the circulation leading to vascular collapse and progressive multiple organ failures, which may lead to death [9]. Currently, there is no effective treatment against ricin intoxication [10].

The long-standing idea about the potency of ricin is that one single molecule of cytosolic RTA is sufficient to kill a cell by inactivating 1500 ribosomes per minute [11]. Recent studies have revealed that a cellular recovery mechanism counteracts sublethal AB toxin intoxications, suggesting that a host protective mechanism may be induced after ricin intoxication [12]. Moreover, a systematic mammalian genetic interaction map suggests that autophagy is one of the factors determining cell susceptibility to ricin [13]. Autophagy is a cellular homeostatic process by which cytoplasmic components including organelles, as well as invading microbes, are engulfed by double-membrane vesicles and targeted for degradation by fusion with late endosomes/lysosomes [14]. Under basal conditions, this process plays a housekeeping role in the elimination of damaged components in the cell so as to maintain nutrient and organelle homeostasis. Therefore, by providing nutrients and energy, autophagy is essential for cell survival. It is also a protective mechanism that allows cells to survive in response to various types of stresses, as well as intracellular pathogens. Autophagy can be cytotoxic by activating other death pathways or by leading to autophagy-dependent cell death (ADCD), which depends on the autophagy machinery rather than that of the canonical cell death pathways [15].

There is growing evidence showing that the intoxication of bacterial toxins interacts with the autophagy pathway with different outcomes [16]. Autophagy functions as a pro-survival or pro-death mechanism depending on the toxin. Anthrax lethal toxin from *Bacillus anthracis* activates autophagy in mammalian cells and autophagy inhibition accelerates toxin-induced cell death. This suggests a protective role of autophagy against anthrax lethal toxin [17]. In contrast, Toxin B from *Clostridium difficile* elicits a strong autophagy response, which contributes to the inhibition of cell proliferation [18]. In this case, inhibition of autophagy attenuates the cytotoxic effect of Toxin B. Similarly, Shiga-like toxin 2 (Stx-2) induces autophagy activation via the ER-stress pathway in intestinal epithelial cells, which leads to cell death [19]. Ricin and Shiga toxins are both protein synthesis inhibitors that rely on similar host cell machinery for internalization, retrograde trafficking from endosomes to the ER and translocation into the cytosol to inhibit protein synthesis. Moreover, a cross-talk between protein synthesis and autophagy is coordinated via mTORC1 (mammalian target of rapamycin complex 1) [20]. These observations prompted us to explore the existence of an autophagic response in ricin-intoxicated cells. In this study, we demonstrate that cell protection against ricin is associated with autophagic degradation; thus, ricin cytotoxicity is aggravated in autophagy-deficient cells. Moreover, the autophagy inducer SMER28 (Small Molecule Enhancer of Rapamycin 28) increases cell survival upon ricin intoxication. 

## 2. Results

### 2.1. Autophagic Degradation Is Involved in Ricin Intoxication

**Figure 1:**
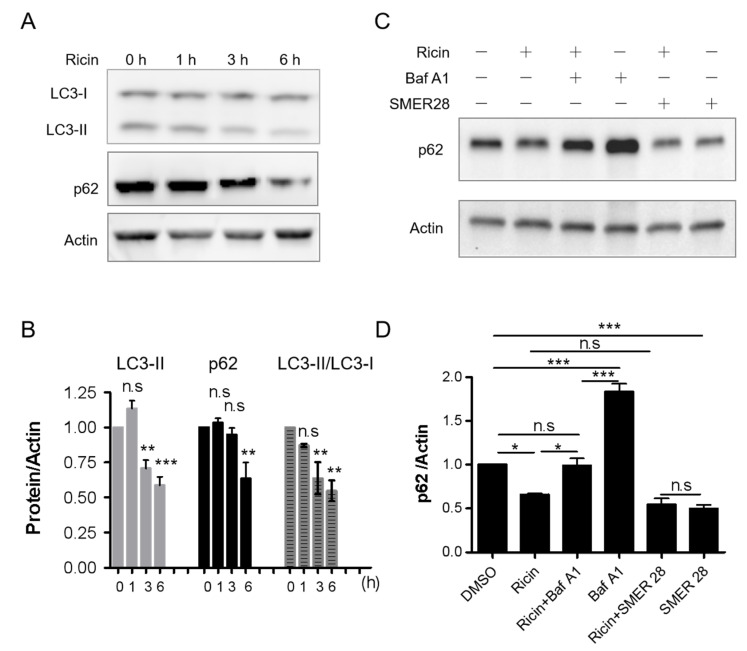
Ricin intoxication modulates LC3-II and p62 levels, which can be reversed by bafilomycin A1 (Baf A1), an autophagy inhibitor. A549 cells were treated with 2 nM ricin for the indicated times. Cell lysates were examined for LC3 and p62 levels by immunoblotting. Actin was used as the loading control (**A**,**C**). (**B**) Quantification of LC3-II and p62 normalized to actin as well as the ratio of LC3-II/LC3-I, corresponding to panel A, is shown as a histogram (mean ± S.E.M, *n* = 4). Statistical significance compared with 0 h was determined using one-way ANOVA with Dunnett’s multiple comparison test, (n.s *p* > 0.05; ** *p* < 0.01; *** *p* < 0.001). (**C**) A549 cells were incubated with 2 nM ricin in the presence or absence of 100 nM Baf A1 or 45 μM SMER28 for 6 h. (**D**) The graph corresponding to panel C is presented as the mean ± S.E.M of 3 experiments. Statistical significance was determined using one-way ANOVA with Tukey’s multiple comparison test, (n.s *p* > 0.05; * *p* < 0.05; *** *p* < 0.001).

The human carcinoma alveolar basal epithelial cell line A549 was chosen to study autophagy in response to ricin intoxication because pulmonary intoxication by aerosol is the most feared scenario [21]. Lipidated LC3 protein (microtubule-associated protein 1 light chain 3), required for autophagosome formation, was used to monitor autophagy as a bona fide marker [14]. Cytosolic LC3-I is processed into lipidated LC3-II, allowing it to be inserted into the autophagosome membrane during formation. SQSTM1/p62, as an autophagic substrate, is incorporated into completed autophagosomes and then degraded in autolysosomes. Ricin toxin was previously shown to enter cells through receptor-mediated endocytosis and reach the cytosol in less than 1 h [22]. Thus, to study the impact of ricin on autophagy, A549 cells were lysed after 1, 3 and 6 h of intoxication and autophagy markers were detected by immunoblotting. As shown in Figure 1A,B, LC3-II and p62 significantly decreased after 6 h of ricin exposure. After 18 h of exposure to ricin, there was also a substantial decrease in LC3-II and p62 that correlated with the dose of toxin (Figure S1). A decrease in the level of LC3-II could be due to either inhibition of autophagy initiation or induction of autophagic degradation. However, the concomitant reduction of p62 implies that the decrease in LC3-II was due to enhanced autophagic degradation. Importantly, LC3-I levels were maintained, while the ratio of LC3-II/LC3-I decreased significantly (Figure 1A,B), suggesting that autophagic degradation occurs during ricin intoxication.

To better understand the dynamics of the ricin-related autophagic process, we compared in parallel the ricin-induced changes to the LC3-II/LC3-I ratio and autophagy substrate p62 with those induced by the autophagy degradation inhibitor bafilomycin A1 (Baf A1) and the autophagy inducer SMER28. Baf A1 inhibits autophagic degradation by targeting the lysosomal V-ATPase, thus blocking the endolysosomal acidification, leading to p62 accumulation [23]. Moreover, Baf A1 increased CHO cells’ sensitivity to ricin [24]. SMER28 has been shown to activate autophagy and enhance the clearance of autophagic substrates independently of rapamycin [25]. As expected, Baf A1 significantly increased p62 protein levels (Figure 1D, *n* = 3, *** *p* < 0.001), as well as the LC3-II/LC3-I ratio (Figure S2), compared with non-treated cells. Remarkably, ricin-induced p62 degradation was significantly inhibited by Baf A1(* *p* < 0.05), and ricin-induced variation of the LC3-II/LC3-I ratio was reversed to the level of Baf A1 individually induced level (ns, *p* > 0.05, Figure S2). SMER28 reduced p62 levels down to a level similar to the extent of ricin-induced p62 degradation. However, ricin did not further reduce the levels of p62 induced by SMER28 (Figure 1C,D), suggesting that the two treatments may result in p62 degradation by a similar pathway.

To explore whether ricin intoxication affects normal cells differently from malignant cells, we monitored autophagy in primary Human Umbilical Vein Endothelial Cells (HUVECs). We observed similar changes in LC3-II and p62 levels as in A549 cells after ricin intoxication at the same concentration for 6 h (Figure S3). Again, ricin-induced degradation of LC3-II and p62 can be reversed by Baf A1 in HUVECs. Taken together, our results demonstrate that ricin intoxication affects autophagic degradation in human cells.

### 2.2. Autophagy Deficiency Increases Ricin Cytotoxicity

**Figure 2:**
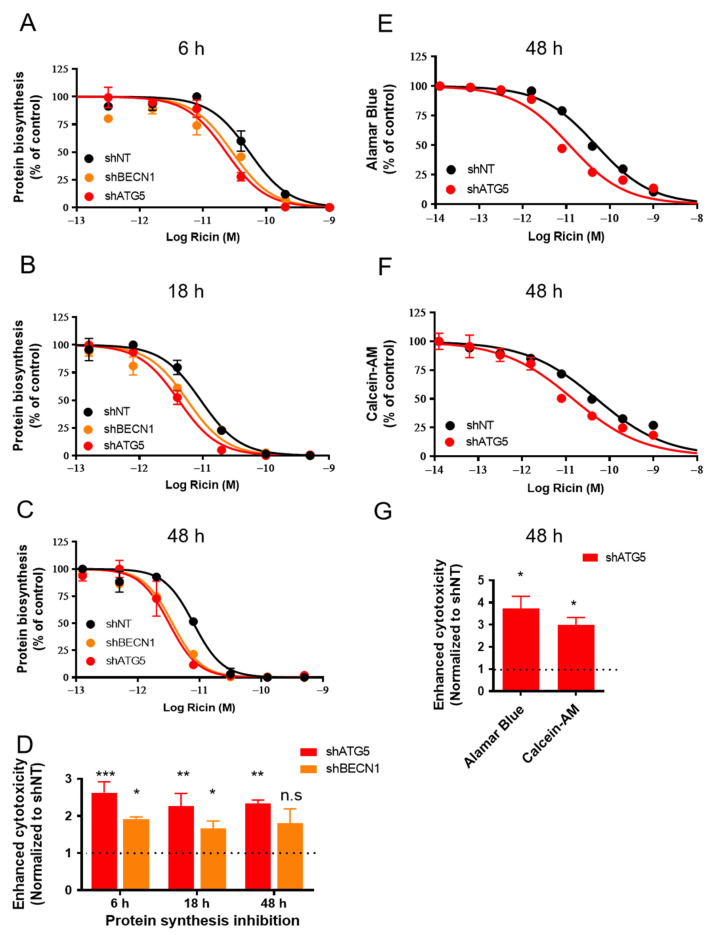
Autophagy-deficient HFF cells are more sensitive to ricin cytotoxicity. shNT, shBECN1 and shATG5 HFF cells were incubated with increasing concentrations of ricin for 6 h (**A**), 18 h (**B**) and 48 h (**C**). The medium was removed and replaced with DMEM containing [^14^C]-leucine at 0.5 μCi/mL for 4 h. Protein synthesis inhibition was measured by scintillation counting as the amount of [^14^C]-leucine incorporated in cells. Each data point represents the mean of duplicate ± S.D. of one representative experiment (*n* = 2~4). (**D**) The histogram shows the mean ± S.E.M of enhanced cytotoxicity as compared with shNT control cells from 2~4 experiments (**A**–**C**). Enhanced ricin cytotoxicity on autophagy-deficient cells was determined by 1/R, and R = IC_50_ (shBECN1 or shATG5)/IC_50_ (shNT). Statistical significance compared with the control was determined using one-way ANOVA with Dunnett’s Multiple Comparison Test (n.s *p* > 0.05; * *p* < 0.05; ** *p* < 0.01; *** *p* < 0.001). Alamar Blue (**E**) and Calcein-AM (**F**) were employed to examine the cell viability of shNT and shATG5 HFF cells after the addition of ricin for 48 h. Each data point represents the mean of duplicate ± S.D of one representative experiment (*n* = 3~4). (**G**) The graph shows the mean ± S.E.M of enhanced cytotoxicity for each assay in (**E**,**F**) (calculated as (**D**)) from 3~4 experiments. Statistical significance compared with control was determined using paired *t*-test, * *p* < 0.05).

To investigate the role of autophagy in ricin intoxication, we used ricin-sensitive human foreskin fibroblasts (HFF) stably expressing shRNA (short hairpin RNA) targeting ATG5 (shATG5) or BECN1/Beclin1 (shBECN1) [26]. ATG5 forms a complex with ATG12 and 16L1, which is necessary for LC3-I to be conjugated to phosphatidylethanolamine (PE) to form LC3-II [27]. Beclin 1 and its complex are involved in phagophore formation and autophagosome maturation [28]. Knocking down these essential autophagy proteins leads to autophagy deficiency, which is confirmed by the reduction in the levels of LC3-I and LC3-II [26]. To elucidate the consequence of autophagy on ricin cytotoxicity, shATG5, shBECN1 and shNT control cells (HFF cells expressing non-targeted shRNA) were incubated with ricin for 6 h, 18 h and 48 h. Ricin-induced protein synthesis inhibition was compared for the three cell lines by measuring the incorporation of [^14^C]-labeled leucine into nascent proteins (Figure 2A–C). The results showed that, at each time point, protein synthesis was more inhibited in shATG5 cells (6 h: 2.63 ± 0.29-fold; 18 h: 2.26 ± 0.35-fold; 48 h: 2.35± 0.08-fold) and in shBECN1 cells (6 h: 1.92 ± 0.06-fold; 18 h: 1.67 ± 0.19-fold; 48 h: 1.81 ± 0.38-fold) than in shNT control cells (*n* = 2~4, Figure 2A–D). This indicates that autophagy-deficient cell lines, i.e., shATG5 (6 h: *** *p* < 0.001; 18 h and 48 h: ** *p* < 0.01) and shBECN1 until 18 h (6 h and 18 h: * *p* < 0.05) were significantly more susceptible to ricin cytotoxicity than cells with an intact autophagic pathway.

We then evaluated the cell viability after 48 h of intoxication by ricin using Alamar Blue (Figure 2E) and Calcein-AM assays (Figure 2F). Alamar Blue is a redox indicator that is used to evaluate metabolic functions and cellular health [29]. Calcein-AM is a non-fluorescent, hydrophobic compound that easily permeates intact and living cells, and is hydrolyzed by intracellular esterases to produce Calcein, a hydrophilic and strongly fluorescent compound in the cell cytoplasm [30]. Both viability assays demonstrated that the toxic effect of ricin on cell viability was increased in the autophagy-deficient shATG5 cells (3.74 ± 0.55-fold, * *p* < 0.05, *n* = 4; 3.00 ± 0.33-fold, * *p* < 0.05, *n* = 3) compared with the shNT control cells (Figure 2E–G). Altogether, our data show that autophagy deficiency generated by genetic interventions increases ricin cytotoxicity.

### 2.3. Autophagy Deficiency Attenuates Ricin Degradation

**Figure 3:**
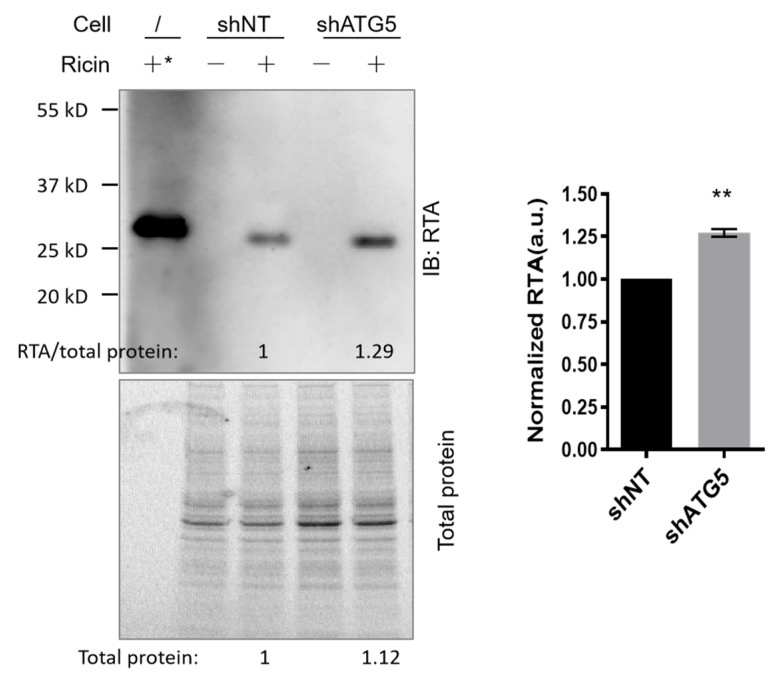
Ricin degradation is reduced in autophagy-deficient HFF cells. shNT and shATG5 HFF cells were incubated with or without ricin (10 nM, 150 ng in 250 μL complete medium per well) for 6 h. Cell lysates were examined for cellular ricin by immunoblot with anti-ricin A (RTA) antibodies. *: Purified ricin protein (20 ng) was used as a molecular weight control. Total proteins of each lysate were imaged and analyzed by the Gel Doc™ EX system (BIO-RAD, Hercules, CA, USA) for use as a loading control. The blot is from one representative experiment. The histogram shows the quantification of RTA normalized to the loading control in shNT and shATG5 cells (mean ± S.E.M, *n* = 3). Statistical significance compared with control was determined using paired *t*-test (** *p* < 0.01).

Autophagy is a conserved and tightly regulated process crucial for cellular homeostasis and survival by degrading cellular components as well as some invading pathogens. Toxins relying on retrograde transport to reach the cytosol from the ER usually have a low lysine content to escape from ubiquitin-mediated proteasomal degradation [31,32]. Considering that autophagy deficiency enhanced ricin cytotoxicity, we questioned whether the protective role of autophagy in ricin intoxication may be related to the autophagy-mediated degradation of ricin. Thus, shNT and shATG5 cells were exposed to ricin (10 nM) for 6 h. Their lysates were then examined for the quantities of intracellular RTA, the enzymatic subunit of ricin responsible for inactivating ribosomes. Immunoblotting of RTA revealed that cellular ricin, normalized to total protein of lysate, was significantly higher in shATG5 cells than in control shNT cells (Figure 3, *n* = 3, *p* = 0.0024). This suggests that autophagy deficiency attenuates ricin degradation.

### 2.4. The Autophagy Inducer SMER28 Increases Cell Survival upon Ricin Intoxication

**Figure 4:**
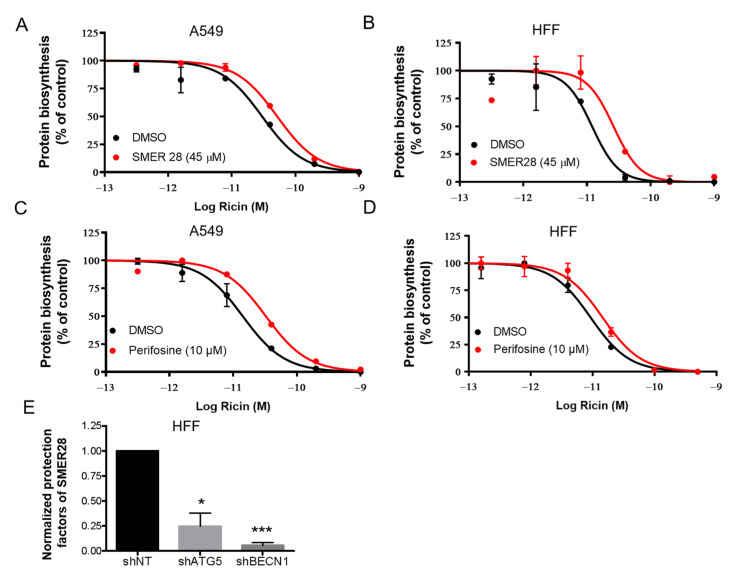
Autophagy inducers protect cells from ricin intoxication. A549 (**A**,**C**) and HFF (**B**,**D**) cells were incubated with SMER28 (**A**,**B**) or perifosine (**C**,**D**) for 1 h at the indicated concentrations and then challenged with increasing doses of ricin for 18 h in the presence of the compounds. A protein synthesis inhibition assay was performed and analyzed as in Figure 2. Each data point represents the mean of duplicate ± S.D. of one representative experiment (*n* = 2). The red curves correspond to the presence of autophagy inducers and the black curves correspond to DMSO vehicle controls. (**E**) HFF shNT, shATG5 and shBECN1 cells were incubated with SMER28 or DMSO vehicle and challenged with ricin as A and B. Normalized protection factors (see Materials and Methods) of SMER28 on shNT, shATG5 and shBECN1 HFF cells are shown as the mean ± S.E.M from 3 separate experiments. The differences to the control (shNT) were compared using paired t-test (* *p* < 0.05, *n* = 3; *** *p* < 0.001, *n* = 3).

As we found that autophagy plays a protective role in ricin intoxication and contributes to ricin degradation, we explored whether autophagy inducers could protect cells against ricin intoxication. We selected two autophagy inducers, SMER28 (an mTOR-dependent autophagy inducer) and perifosine (Akt inhibitor, an mTOR-dependent autophagy inducer) [33], and examined whether they could protect A549 (Figure 4A,C) and HFF cells (Figure 4B,D) challenged by ricin toxin. Protein synthesis inhibition and Alamar Blue assays were used as described previously. Treatment of the cells with SMER28 at 45 μM resulted in a moderate protective effect against ricin in both cell lines (A549: 1.7-fold; HFF: 2.2-fold; Figure 4A,B). Perifosine at 10 μM also induced protection against ricin in both cell lines (A549: 2.2-fold; HFF: 1.6-fold; Figure 4C,D). However, the results were probably underestimated due to its observed toxicity on the cells (Figure S4). 

Then, we tested the effect of SMER28 on autophagy-deficient HFF shATG5 and shBECN1 cells and calculated the protection factor (R) of SMER28 against ricin in each cell line. The protective effect of SMER28 against ricin was significantly decreased in shATG5 cells (* *p* < 0.05, *n* = 3) and shBECN1 cells (*** *p* < 0.001, *n* = 3) compared with that of shNT cells (Figure 4E). These results therefore demonstrate that the reduction in ricin cytotoxicity by SMER28 occurs via autophagy.

**Figure 5:**
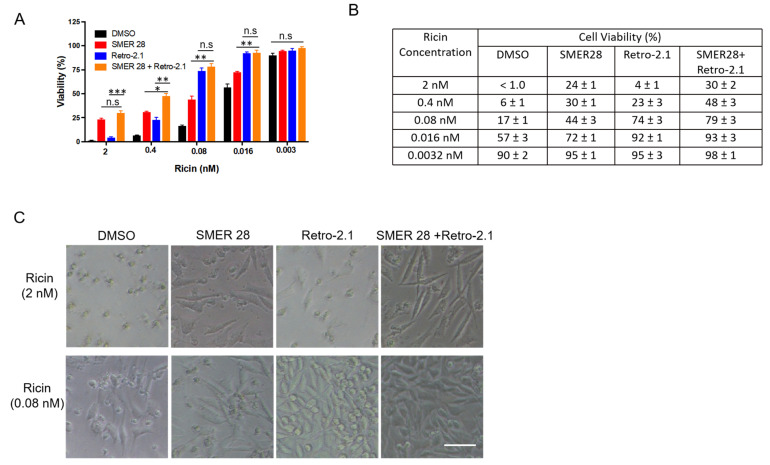
Protective effect of a combination of SMER28 and Retro-2.1 against ricin cytotoxicity. L929 cells were pretreated with SMER28 (45 µM), Retro-2.1 (100 nM) or both for 1 h and then challenged with increasing doses of ricin for 64 h. (**A**) Cell viability was evaluated by the Alamar Blue assay. The graph shows cell viability as the mean ± S.E.M of 3 independent experiments. Statistical significance was determined using one-way ANOVA with Bonferroni’s multiple comparison test (n.s *p* > 0.05; * *p* < 0.05; ** *p* < 0.01; *** *p* < 0.001). (**B**) The table shows the cell viability of each condition in (**A**) as the mean ± S.E.M of 3 independent experiments. (**C**) Representative images were obtained with an inverted microscope (Ti-U, Nikon, Tokyo, Japan) from cells challenged by a high dose (2 nM) or low dose (0.08 nM) of ricin. Scale bar, 100 μm.

Knowing that SMER28 protects cells against ricin by activating autophagy, we subsequently investigated the effect of the combination of SMER28 with Retro-2.1 against ricin. Retro-2.1 is an optimized analog of Retro-2 [34], which inhibits ricin toxin by blocking its retrograde trafficking at the interface of early endosomes and the Golgi apparatus [35]. Because of the different mechanisms of action of SMER28 (induction of its autophagy) and Retro-2.1 (inhibition of intracellular trafficking) on ricin, we questioned whether co-treatment could produce an additive protection against ricin in L929 cells, an immortalized fibroblast cell line, rather than a malignant cell line, where Retro-2.1 exhibits the highest protective effect (EC_50_ = 38.7 nM, see Figure S5) compared with that on A549 cells [34]. We used Retro-2.1 at its maximal effective concentration of 100 nM (Figure S5), as well as the autophagy inducer SMER28 at 45 μM. L929 cells were challenged with increasing doses of ricin in the presence of Retro-2.1, SMER28 or both at their fixed concentration for 64 h. Cell viability was evaluated by the Alamar Blue assay (Figure 5A). At the highest dose of ricin (2 nM ricin without drug, cell viability: <1%), the combination of Retro-2.1 and SMER28 increased viability to 30 ± 2%, while cell viability in the presence of Retro-2.1 or SMER28 alone was 4 ± 1% (*** *p* < 0.001) and 24 ± 1%, respectively (n.s *p* > 0.05) (*n* = 3). At a five-fold lower dose (0.4 nM ricin without the drug, cell viability: 6 ± 1%), the combination significantly increased viability to 48 ± 3% (Retro-2.1: 23 ± 3%, * *p* < 0.05; SMER28: 31 ± 1%, ** *p* < 0.01; *n* = 3). Retro-2.1 was more effective against low doses of ricin (see 0.08 nM ricin, Figure 5A–C), while SMER28 was more potent in response to high doses of ricin (see 2 nM and 0.4 nM ricin, Figure 5A–C). Moreover, the combination effect of the two drugs against ricin cytotoxicity was clearly visible on cell morphology (Figure 5B). Ricin intoxicated cells appeared rounded at 0.08 nM ricin. The cells partially retained their fusiform shape characteristic of fibroblasts in the presence of one or the other inhibitor. In the presence of both inhibitors, the cells had a normal appearance: fusiform before cell confluence in the condition of a high dose of toxin and more polygonal at the confluence in the condition of a low dose of toxin. Moreover, both inhibitors and their combination had no apparent toxicity on L929 cells (Figure S6).

Altogether, the combination of Retro-2.1 with SMER28 further enhanced the efficacy of protection of each molecule alone, especially against high doses of ricin, suggesting that these two molecules inhibit ricin cytotoxicity by distinct mechanisms.

## 3. Discussion

Our results suggest that ricin intoxication is associated with autophagic degradation in mammalian cells, which mediates a protective role against ricin cytotoxicity (Figure S7). This hypothesis is supported by the following pieces of evidence: (i) Ricin intoxication induced a time- and dose-dependent decrease in the protein levels of LC3-II, a specific marker of autophagosomes, and p62, a substrate marker of autophagic degradation. These decreases can be reversed by Baf A1 (Figure 1 and Figure S2). (ii) Ricin abundance and its cytotoxicity were increased in autophagy-deficient cells (Figure 2 and Figure 3). (iii) The autophagy inducer SMER28 (and perifosine, despite its toxicity to cells) moderately protected cells against ricin (Figure 4A,B and Figure 5), an effect that was significantly decreased in autophagy-deficient cells (Figure 4E), while autophagy inhibitors induced sensitization to ricin [24].

Under certain circumstances, for example, in neuronal cells in the context of Parkinson’s disease, p62 can be partially degraded by the proteasome [36], but it is primarily degraded during selective autophagy [37]. In Figure 1, we observed that LC3-II and p62 were both decreased because of ricin intoxication. The addition of Baf A1 blocked the autophagic degradation and restored p62 to the level of non-toxin control, suggesting that the p62 decrease was highly associated with autophagy, and an autophagic flux was induced under ricin treatment (Figure 1D). The combination of SMER28 and ricin did not induce additional or synergistic effects on p62 degradation (Figure 1D), suggesting that they both induced a p62 decrease via a common pathway; otherwise, the proteasomal degradation-induced reduction in p62 would be amplified by autophagy-induced p62 degradation. Moreover, Stx-2 induced LC3-II upregulation and autophagic cell death despite inhibiting protein synthesis, similar to ricin [19], suggesting that autophagy markers are stable and independent of toxin-induced protein synthesis inhibition.

Ricin enters the cell through different endocytic processes, allowing the toxin to reach almost all cellular compartments and to depurinate different nucleic acid substrates with multiple lethal consequences [3]. Thus, we used three different approaches to explore the consequences of ricin intoxication on autophagy-deficient cells. The protein synthesis inhibition assay can directly monitor the mechanism of action of ricin by measuring the incorporation of radioactive amino acids. Alternatively, Alamar Blue and Calcein-AM assays were used to evaluate cell viability after ricin intoxication. Ricin cytotoxicity was significantly increased in shATG5 cells at each tested time point, and until 18 h of exposure for shBECN1 cells, suggesting a protective role of autophagy in ricin intoxication (Figure 2). The possible reason for the more moderate effect observed in shBECN1 cells compared with shATG5 cells may be related to the silencing efficiency of BECN1 compared with ATG5 in these cell lines. Indeed, the protein extinction level is about 60% for BECN1 and 80% for ATG5 determined by immunoblotting [26].

The detailed protective mechanism of autophagy in response to ricin remains unclear. Bassik et al. showed that depletion of WDR11 or BECN1, two regulators of autophagy, caused an increase in total cellular RTA in K562 cells [13]. This observation is consistent with ours, as knocking down ATG5 led to enhanced intracellular RTA in HFF cells (Figure 3). Notably, protective autophagy can be represented by the degradation of aggregated proteins, damaged mitochondria or invading pathogens, and it could also contribute to stress protection by fine tuning physiological pathways through the degradation of specific proteins [38].

At least two explanations may account for ricin stimulation of autophagic degradation. Protein biosynthesis inhibition by RTA may stimulate bulk autophagy by random sequestration and degradation of cytosolic material to provide small molecules, especially amino acids for protein synthesis. Alternatively, after retrotranslocation from the endoplasmic reticulum to the cytosol, RTA may be specifically recognized by autophagy adaptors (e.g., p62) as autophagic cargo and targeted to autophagosomes for degradation. p62 can be involved in the selective autophagic clearance of not only ubiquitylated substrates but also certain types of nonubiquitylated substrates [39]. With only two lysine residues, RTA escapes ubiquitination and ERAD [31,32]. Thus, it would be interesting to explore whether RTA is targeted by p62 or other adaptors towards autophagic degradation and whether this procedure is ubiquitin-dependent. Moreover, ricin triggers apoptosis, mainly in cancer cells, via a mechanism involving Caspase3/7 activation, PARP cleavage and DNA fragmentation, thus leading to cell death [40,41]. It would be worthwhile to elucidate the association of protective autophagy with other stress mechanisms, such as apoptosis.

SMER28, a small-molecule enhancer of rapamycin (SMER), identified by chemical screening, was shown to induce mTOR-independent autophagy and to reduce mutant Huntington aggregates and A53T α-synuclein in cellular models of Huntington’s and Parkinson’s disease [25]. In our study, SMER28 was able to protect several mammalian cell lines (A549, HFF and L929 cells) against ricin toxin. The potential of SMER28 as a therapeutic anti-ricin drug alone may be limited. However, the combination of Retro-2.1 and SMER28 significantly increased cell viability at the high dose of ricin compared with each drug alone (Figure 5A), indicating that autophagy regulators may have potential for therapeutic intervention, for example as a complement to retrograde transport inhibitors such as Retro-2.1 or antitoxin antibodies, as well as anti-inflammatory drugs. 

Several therapeutic approaches against ricin intoxication are being developed [42]. Neutralizing antibodies have shown some success in animal models, but need to act before the toxin enters the cells [43,44]. Molecules targeting the enzymatic activity of ricin have never succeeded in animal models [42,45]. Retrograde trafficking inhibitors have shown promising results in mice [35]. Nevertheless, it is well-admitted that ricin intoxication is very difficult to treat in vivo. Thus, multiple combined therapies based on different antitoxin mechanisms may be needed to reach the goal [35,46,47]. Our present work demonstrates that autophagic degradation contributes to ricin degradation and that stimulation of autophagic degradation may help to counteract ricin intoxication.

## 4. Materials and Methods

### 4.1. Materials and Reagents

The following products were purchased from the indicated commercial sources: l-[^14^C(U)]-leucine was purchased from Perkin-Elmer (Waltham, MA, USA); DMSO (D4540), bafilomycin A1 (B1793), gelatin (G7765), anti-LC3B (L7543), anti-p62 (P0067) and Calcein-AM (17783) were purchased from Sigma (St. Louis, MO, USA); Alamar Blue™ Cell Viability Reagent (DAL1025) was purchased from ThermoFisher Scientific (Rockford, IL, USA); anti-actin (ab3280) was purchased from Abcam; anti-RTA (SC-52190) was purchased from Santa Cruz Biotechnology (Santa Cruz, CA, USA). Ricin toxin was a kind gift from Dr. Bruno Beaumelle, Institut de Recherche en Infectiologie de Montpellier, France.

### 4.2. Cell Culture and Ricin Intoxication Assays

#### 4.2.1. Autophagy-Deficient HFF Cells

Stable HFF cell lines deficient in autophagy were established using lentiviral transduction [26]. ATG5, BECN1 and non-target shRNA were purchased from Sigma (MISSION pLKO.1-puro shRNA lentiviruses). After transduction, shATG5-, shBECN1- and shNT-expressing HFF were selected by the addition of puromycin to the culture medium (5 μg/mL, InvivoGen, San Diego, CA, USA). 

#### 4.2.2. Evaluation of Protein Biosynthesis Inhibition

For detailed experimental procedures, see a previous description [48]. Briefly, cells were grown in Cytostar T scintillating microplates (Perkin Elmer, MA, USA) with incorporated scintillators. Cells in the presence of the indicated compounds (or 0.1% DMSO) were challenged with increasing doses of ricin. After incubation for the indicated time, the medium was removed and replaced with DMEM without leucine (Eurobio) containing 10% fetal bovine serum, 2 mM L-glutamine, 0.1 mM non-essential amino acids, and 1% penicillin/streptomycin supplemented with 0.5 μCi/mL [^14^C]-leucine. The cells were grown for an additional 4 h at 37 °C, and then protein biosynthesis was determined by measuring the incorporation of radiolabeled leucine into cells using a Wallac 1450 MicroBeta liquid scintillation counter (Perkin-Elmer). The mean percentage of protein biosynthesis was determined and normalized from duplicate wells. All values were expressed as the mean ± S.E.M. Data were fitted with Prism 7 software (Graphpad Inc., San Diego, CA, USA) to obtain the 50% inhibitory toxin concentration (IC_50_), i.e., the concentration of toxin that is required to inhibit 50% of cellular protein biosynthesis. IC_50_ values and protection factor R (R = IC_50_ (drug)/IC_50_ (DMSO)) were determined by the software’s nonlinear regression “dose-response EC_50_ shift equation”. The goodness of fit for ricin alone (carrier) and with drug/treatment was assessed by r^2^ and confidence intervals. 

Enhanced ricin cytotoxicity on autophagy-deficient cells was determined by 1/R, and R = IC_50_ (shBECN1 or shATG5)/IC_50_ (shNT).

#### 4.2.3. Alamar Blue and Calcein-AM Assays

Cells seeded in Corning 96-well Flat Clear Bottom Black Microplates (#3603) were incubated with increasing concentrations of ricin in complete medium for the indicated time. The medium was replaced with fresh complete medium including Alamar Blue (final concentration 1:10). Then, the fluorescence (540/590 nm) was measured 1 h later by a Cytation^TM^ 5 cell imaging multi-mode reader (BioTek, Winooski, VT, USA). Calcein-AM in PBS (1: 2000, 5 µM) was incubated with cells for 30 min and its intracellular fluorescence (495/520 nm) was measured by Cytation 5.

### 4.3. Immunoblot Analysis

Cells were lysed in M-PER^®^ Mammalian Protein Extraction Reagent (78503, Thermo Scientific, Waltham, MA, USA) and lysates supplemented with 4× Laemmli sample buffer (161-0747, BIO-RAD, Hercules, CA, USA) were heated at 95 °C for 10 min. Protein extracts were resolved on SDS-PAGE gels and electrotransferred onto a PVDF membrane (BIO-RAD). Membranes were incubated in blocking buffer (5% non-fat dry milk in PBS) and then probed with primary antibodies overnight. HRP-labeled antibodies were used as a secondary antibody and revealed by Image Quant™ LAS 4000 systems (GE healthcare, Chicago, IL, USA). Immunoblotted actin or total proteins imaged by the GelDoc™ EZ system (BIO-RAD) were used to quantify protein loading.

## Data Availability

Most data is contained within the Section 2 of this article. Additional data are available on request from the corresponding author.

## Supplementary Materials

The following supporting information can be downloaded at: https://www.mdpi.com/article/10.3390/toxins15050304/s1, Figure S1: Ricin intoxication modulates autophagy by decreasing LC3 and p62 levels in a dose-dependent way; Figure S2: Ricin intoxication (6 h) modulates autophagy in A549 cells by decreasing the ratio of LC3-II/LC3-I, which can be reversed by Baf A1, related to Figure 1C,D; Figure S3: Ricin intoxication modulates autophagy in HUVECs cells, related to Figure 1C; Figure S4: Toxicity of SMER28 and perifosine; Figure S5: Activity of Retro-2.1 against ricin in L929 cells; Figure S6: SMER28, Retro-2.1 and their combination did not affect the viability of L929 cells; Figure S7: Model depicting that autophagic degradation is involved in cell protection against ricin toxin.
