# Supplementary material for: Autophagic Degradation Is Involved in Cell Protection against Ricin Toxin
Source: Toxins (Basel). 2023 Apr 23;15(5):304. doi: 10.3390/toxins15050304 (PMC10222476; doi:10.3390/toxins15050304)
Supplement: Supplementary file 1 [file toxins-15-00304-s001.zip › toxins-2267945-supplementary.pdf]

Supplementary Matrials

Autophagic Degradation Is Involved in Cell Protection against Ricin Toxin

Yu Wu [1,2], Clémence Taisne [3], Nassim Mahtal [1], Alison Forrester [4], Marion Lussignol [3], Jean-Christophe Cintrat [5], Audrey Esclatine [3], Daniel Gillet [1,*] and Julien Barbier [1,*]

[1] Service d'ingénierie moléculaire pour la santé (SIMoS), Médicaments et Technologies pour la Santé (DMTS), Université Paris-Saclay, CEA, INRAE, Gif-sur-Yvette 91191, France; wuyu@ustc.edu.cn (Y.W.); n.mahtal@gmail.com (N.M.)

[2] Institute of Immunology, The First Affiliated Hospital of USTC, Division of Life Sciences and Medicine, University of Science and Technology of China (USTC), Hefei 230001, China

[3] Institute for Integrative Biology of the Cell (I2BC), Université Paris-Saclay, CEA, CNRS, Gif-sur-Yvette 91191, France; clemence.taisne@u-psud.fr (C.T.); marion.lussignol@u-psud.fr (M.L.); audrey.esclatine@u-psud.fr (A.E.)

[4] Research Unit of Biochemistry and Cell Biology (URBC), Namur Research Institute for Life Sciences (NARILIS), University of Namur, 5000 Namur, Belgium; alison.forrester@unamur.be

[5] Service de Chimie Bio-organique et Marquage (SCBM), Médicaments et Technologies pour la Santé (DMTS), Université Paris-Saclay, CEA, INRAE, Gif-sur-Yvette 91191, France; jean-christophe.cintrat@cea.fr

[*] Correspondence: daniel.gillet@cea.fr (D.G.); julien.barbier@cea.fr (J.B.); Tel.: +33-169087646, Fax: + 33-16908907 (D.G. & J.B.)

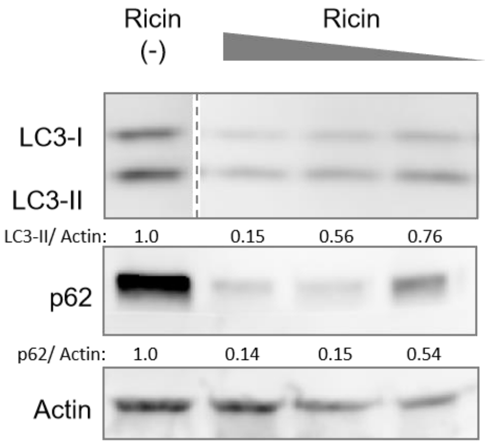

Figure S1. Ricin stimulates autophagy in a dose-dependent manner. A549 Cells were treated with ricin at 2 nM, 0.67 nM, 0.22 nM for 18 h. Then, cell lysates were examined for LC3 or p62 levels by immunoblot. Actin was used as the loading control. Quantification of LC3-II and p62 normalized to actin is shown under the blots from one representative experiment. Immunoblot of LC3 from single experiment was spliced to rearrange the order of samples.

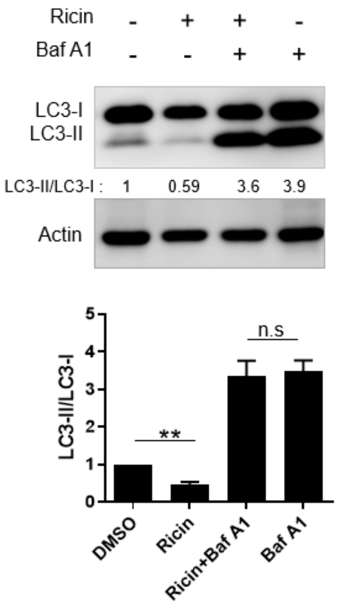

Figure S2. Ricin intoxication (6h) modulates autophagy in A549 cells by decreasing the ratio of LC3-II/LC3-I, which can be reversed by Baf A1, related to Fig 1C and 1D. The ratio of LC3-II/LC3-I is shown in the histogram, presented as mean ± S.E.M of 3 experiments (**, $p < 0.01$; n.s, $p > 0.05$).

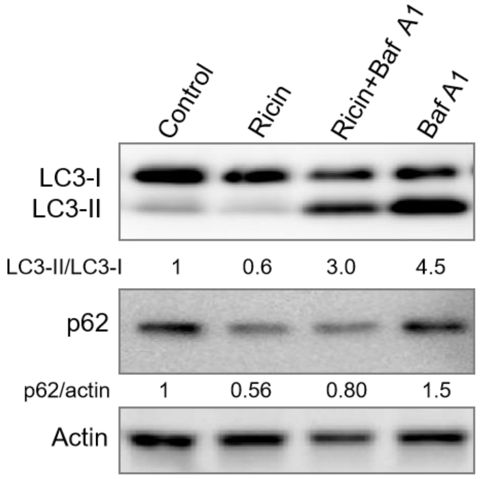

Figure S3. Ricin intoxication modulates autophagy in HUVECs, related to Fig 1C. Quantification of LC3-II/ LC3-I and p62 normalized to actin is shown under the blots.

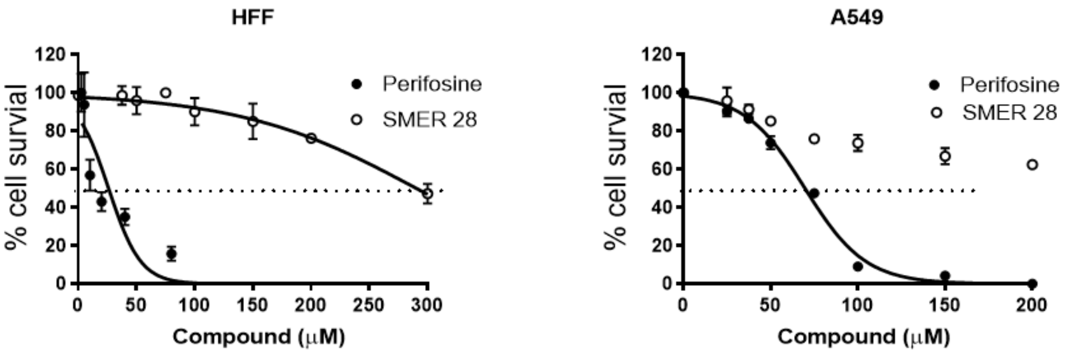

Figure S4. Toxicity of SMER28 and perifosine. HFF or A549 cells were incubated with compounds at the indicated concentrations for 24 h, their viability was measured by Alamar blue. Data are shown as mean values of duplicate wells ± S.D. from one representative experiments (n = 2).

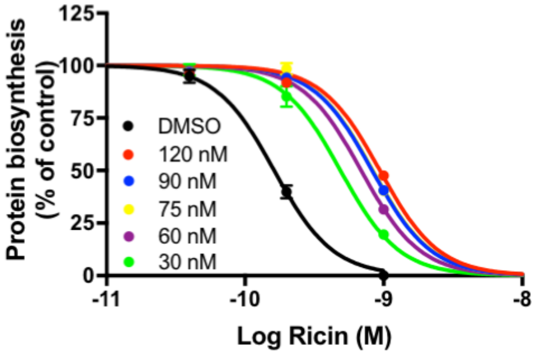

Figure S5. Activity of Retro-2.1 against ricin in L929 cells. Cells were respectively incubated with Retro-2.1 at the indicated concentrations for 1 h and then challenged with increasing doses of ricin for 18 h in the presence of compounds. Protein synthesis inhibition assay was performed and analyzed as shown in Figure 2. Each data point represents the mean of duplicates ± S.D. of one representative experiment (*n* = 2).

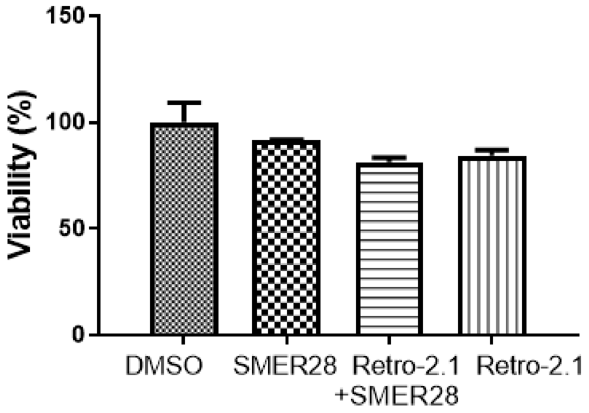

Figure S6. SMER28, Retro-2.1 and their combination have no apparent toxicity on L929 cells. L929 cells were treated with compounds as in Figure 5, cell viability was analysed by Calcein-AM assay. Each column represents the mean of duplicate wells ± S.D. of a representative experiment (*n* = 3).

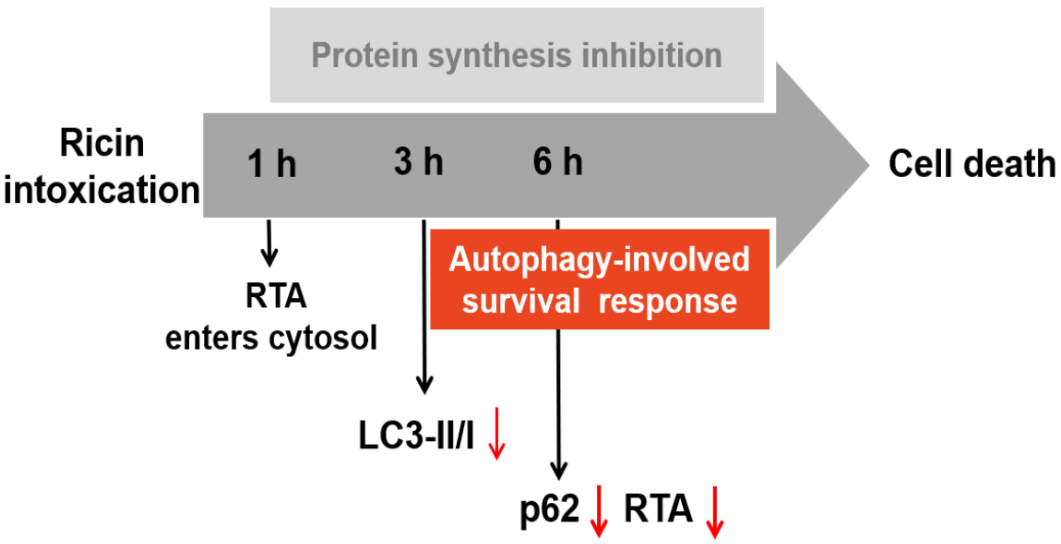

Figure S7. Model depicting that Autophagic degradation is involved in cell protection against ricin toxin.
